# How are we measuring physical activity and sedentary behaviour in the four home nations of the UK? A narrative review of current surveillance measures and future directions

**DOI:** 10.1136/bjsports-2018-100355

**Published:** 2019-05-22

**Authors:** Tessa Strain, Karen Milton, Philippa Dall, Martyn Standage, Nanette Mutrie

**Affiliations:** 1 MRC Epidemiology Unit, University of Cambridge, Cambridge, UK; 2 Norwich Medical School, University of East Anglia, Norwich, UK; 3 School of Health and Life Sciences, Glasgow Caledonian University, Glasgow, UK; 4 Centre for Motivation and Health Behaviour Change, Department for Health, University of Bath, Bath, UK; 5 Physical Activity for Health Research Centre, Moray House School of Education, University of Edinburgh, Edinburgh, UK

**Keywords:** surveillance, physical activity, sedentary behaviour, questionnaires, measurement

## Abstract

**Background/objectives:**

To explore and describe the comparability between the surveys of the UK home nations (England, Northern Ireland, Scotland, Wales) that monitor compliance with the Chief Medical Officers’ physical activity (PA) recommendations. We also suggest ways to improve the UK national PA and sedentary behaviour (SB) surveillance systems.

**Methods:**

We identified national surveys that monitor PA and SB through searching UK-wide and devolved administration websites, the Global Observatory for Physical Activity Country Cards and the Active Healthy Kids Report Cards. Subsequently, we extracted information from survey documentation on the survey commissioners and contractors, method of administration, current questionnaire details relevant to the PA recommendations, questionnaire changes over the previous decade and the most recent prevalence figures.

**Results:**

For adults and older adults, five surveys assess the moderate-to-vigorous PA (MVPA) recommendation, three assess muscle strengthening and three assess SB. For older adults only, three assess balance and co-ordination. For children, seven assess MVPA, none assess muscle strengthening and five assess SB. Only one survey reports on the under 5 PA recommendation. There is no part of the recommendations for which comparable estimates can be calculated across all four home nations. The greatest variation is among the SB questions and reporting. No survey has regularly used device-based measures.

**Conclusion:**

UK surveillance of the PA recommendations is complex, undertaken separately in the home nations, using multiple surveys that cover adults and children separately. We recommend that the costs and benefits of harmonising the existing questionnaires are considered, along with the potential introduction of device-based measures.

## Introduction

Physical activity (PA) reduces the risk of premature death from the leading non-communicable diseases including heart disease, stroke, diabetes and certain types of cancer.^1 2^ Leading a physically active lifestyle has many psychological and cognitive benefits including a reduced risk of dementia, lowered risk of depression and improved well-being.^1^ A dose–response relationship exists between PA and health—higher levels of activity are associated with greater health benefits, although the relationship is non-linear.^1 2^

National PA recommendations provide consensus on the amount, intensity, frequency and type of PA needed to improve health and reduce the risk of non-communicable diseases.^3^ These recommendations are typically based on comprehensive systematic reviews of the best available scientific evidence linking PA to a range of health outcomes (eg,^1 2^).

In 2011, the Chief Medical Officers (CMOs) commissioned a review of the PA recommendations in England, Wales, Scotland and Northern Ireland (the four ‘home nations’), which led to the first common set of PA recommendations across the UK.^4^ These recommendations emphasised the importance of PA across the life course, with separate guidance for early years (under 5s), children and adolescents, adults and older adults. In light of increasing evidence that high levels of sitting may have deleterious health effects,^5^ these guidelines also included a statement on sedentary behaviour (SB). The benefits of PA that strengthens muscles and improves balance beyond health ageing were also acknowledged, and were given greater prominence compared with previous home nations’ policy documents.^4^ Summarised in box 1 are the 2011 PA recommendations for each age group.

Box 1 **The 2011 physical activity recommendations for the UK^4^**

**Early years (under 5s)**

- PA should be encouraged from birth, particularly through floor-based play and water-based activities in safe environments.
- Children of preschool age who are capable of walking unaided should be physically active daily for at least 180 min (3 hours), spread throughout the day.
- All under 5s should minimise the amount of time spent being sedentary (being restrained or sitting) for extended periods (except time spent sleeping).

**Children and young people (5–18 years)**

- All children and young people should engage in moderate to vigorous intensity PA for at least 60 min and up to several hours every day.
- Vigorous intensity activities, including those that strengthen muscle and bone, should be incorporated at least 3 days a week.
- All children and young people should minimise the amount of time spent being sedentary (sitting) for extended periods.

**Adults (19–64 years)**

- Adults should aim to be active daily. Over a week, activity should add up to at least 150 min (2½ hours) of moderate intensity activity in bouts of 10 min or more. Alternatively, comparable benefits can be achieved through 75 min of vigorous intensity activity spread across the week or a combination of moderate and vigorous intensity activity.
- Adults should also undertake PA to improve muscle strength on at least 2 days a week.
- All adults should minimise the amount of time spent being sedentary (sitting) for extended periods.

**Older adults (65+ years)**

- Older adults who participate in any amount of PA gain some health benefits, including maintenance of good physical and cognitive function. Some PA is better than none, and more PA provides greater health benefits.
- Older adults should aim to be active daily. Over a week, activity should add up to at least 150 min (2½ hours) of moderate intensity activity in bouts of 10 min or more—one way to approach this is to do 30 min on at least 5 days a week. For those who are already regularly active at moderate intensity, comparable benefits can be achieved through 75 min of vigorous intensity activity spread across the week or a combination of moderate and vigorous activity.
- Older adults should also undertake PA to improve muscle strength on at least 2 days a week.
- Older adults at risk of falls should incorporate PA to improve balance and co-ordination on at least 2 days a week.
- All older adults should minimise the amount of time spent being sedentary (sitting) for extended periods.

In 2018, the CMOs appointed a number of Expert Working Groups to update these recommendations. For the first time, an Expert Working Group on Communication and Surveillance, including the authors, was commissioned to review the implications for surveillance.^6^ Conducting national surveillance on PA is important for benchmarking current activity levels among populations, setting targets and monitoring progress over time.^3 7^ National surveillance enables participation of the home nations in global initiatives such as the Global Observatory for Physical Activity (Go-PA!) Country Cards and large cross-national academic studies.^8 9^ To date, no analysis has explored the similarities and differences between the home nations’ surveys and how appropriate each survey is for assessing PA prevalence against the UK recommendations. Such an analysis is critical ahead of the publication of the 2019 recommendations^10^ to inform potential changes to the surveillance of PA and SB in the UK and ensure consistency across the home nations. Specifically, our aim was to review the PA and SB-related questions in the four UK home nations’ surveillance systems to:

- Determine if and how they address each component of the UK recommendations;
- examine the nature of surveillance systems in terms of using questionnaires or device-based measures;
- Examine the comparability of estimates obtained from each surveillance system;
- Suggest improvements to strengthen national PA and SB surveillance systems.

## Methods

In January 2018, the CMOs appointed the authors to the Expert Working Group on Communication and Surveillance as part of the update of the UK PA recommendations. The remit was to describe the current PA and SB surveillance methods to inform decision-making and ensure the systems used are appropriate and can be aligned with the new recommendations. National surveys of PA and SB prevalence were identified (January–March 2018, updated October 2018) by searching UK-wide and devolved administration websites, the home nations’ Go-PA! Country Cards^9^ and their Active Healthy Kids Report Cards.^11–14^ Inclusion criteria were that the survey (1) reports on prevalence of one or more, but not necessarily all, of the 2011 UK PA recommendations; (2) is nationally representative at a home nation or UK-wide level; and (3) has plans to re-collect data using comparable methods. Cohort studies were excluded as they track the same individuals over time and are thus inappropriate for population health surveillance. Recent results, data documentation and technical reports were obtained for surveys that met the inclusion criteria. These were located on the survey websites, UK Government/devolved administration websites and the UK Data Archive (specific references provided in the Results section).

The following information was extracted and summarised in tables for each recommendation: survey commissioners and contractors; method of administration; details of the current questionnaire relevant to the PA and SB recommendations; changes in the questionnaire over the previous decade; the most recent figures that describe the percentages of the population meeting the current recommendations. Questions relating to SB were categorised according to the TAxonomy of Self-reported Sedentary behaviours Tools (TASST) framework.^15^ A narrative review was undertaken to explore differences between the survey methods.

## Results

### Overview of surveys

Table 1 presents the details of the UK surveys that have been used to monitor trends in the percentages of the population meeting one or more of the PA or SB recommendations. The Government/devolved administration departments usually commission the surveys, which are contracted out to social research companies. Most are interviewer-led computer-assisted personal interviews: Health Survey for England (HSE),^16^ Health Survey for Northern Ireland (HSNI),^17^ Scottish Health Survey (SHeS),^18^ Continuous Household Survey (CHS)^19^ and the National Survey for Wales (NSW).^20^ Others are administered by telephone (the Active Lives Survey (ALS)^21^ and the ALS Children and Young People Survey^22^) or various methods of self-administration (Young Persons’ Behaviour and Attitudes Survey (YPBAS)^23^ and the Health Behaviour in School-Aged Children (HBSC)^24–26^ survey).

**Table 1:** Surveys that report on the percentage of the population meeting the UK physical activity guidelines in 2018 by country and the recommendations monitored

**Country**	**Survey**	**Commissioners**	**Contracted to**	**Survey method**	**Recommendation measured**						
					**MVPA**		**Muscle strength**		**Balance**	**Sedentary behaviour**	
					**Adults/older adults**	**Children**	**Adults/older adults**	**Children**	**Older adults**	**Adults/older adults**	**Children**
England	Active Lives Survey^21^	Sport England in partnership with other bodies and government departments	Ipsos MORI	Postal invite to online (mobile/desktop) completion. Paper questionnaire sent if non-response	✓*						
	Health Survey for England^16^	Department of Health	National Centre for Social Research	Computer-assisted personal interviewing	✔*	✔†	✔		✔	✔	✔
	Active Lives: Children and Young People Survey^22^	Sport England in partnership with government departments	Ipsos MORI	School-based, self-administered online. Additional teacher and parent questionnaires		✔					
Northern Ireland	Health Survey for Northern Ireland^17^	Department of Health	Central Survey Unit of the Northern Ireland Statistics and Research Agency	Computer-assisted personal interviewing	✔		✔		✔	✔	
	Young Persons’ Behaviour and Attitudes Survey^23^	Department of Health	Central Survey Unit of the Northern Ireland Statistics and Research Agency	Self-administered personal interviewing (now uses tablets)		✔					‡
	Continuous Household Survey^19^	A number of Government Departments and Agencies	Central Survey Unit of the Northern Ireland Statistics and Research Agency	Computer-assisted personal interviewing		✔					
Scotland	Scottish Health Survey^18^	The Scottish Government	Scottish Centre for Social Research	Computer-assisted personal interviewing	✔	✔	✔		✔	✔	✔
Wales	National Survey for Wales^20^	Welsh Government	Office for National Statistics	Computer-assisted personal interviewing	✔	✔					‡
England, Scotland, Wales	Health Behaviour in School-Aged Children^24–26^	Supported by WHO and other partners	Undertaken by a consortium of academics from different countries	School-based, self-administered questionnaires, varying methods of administration		✔					‡

*Official statistic provided by Active Lives Survey not the Health Survey for England.

†Also reports on recommendation for under 5s separately to children 5–15 years old.

‡Some measure of a specific behaviour or domain, most commonly TV/screen time.

MVPA, moderate-to-vigorous physical activity.

### Measurement of the moderate-to-vigorous physical activity recommendations

#### Adults and older adults

Table 2 presents an overview of the UK surveys’ current questionnaires that assess the percentage of adults and older adults that meet the ‘150 min’ moderate-to-vigorous PA (MVPA) component of the 2011 PA recommendations, and the latest estimates.

**Table 2:** Overview of the moderate-to-vigorous physical activity surveillance methods of the UK national surveys

Country	Survey	Current questionnaire	Latest prevalence estimate
England	Active Lives Survey	4-week recall period Frequency of sessions, duration of average session Walking, cycling, sport and exercise activities, dance, gardening Asked of all ≥16 years	Aged ≥16 years: 62% M: 65% W: 60% (2017–2018)^21^
	Health Survey for England	4-week recall period Frequency of sessions, duration of average session Walking, heavy housework, gardening/manual, sport and exercises, six items on occupational activity Included approximately every 4 years Asked of all aged ≥16 years, data reported for ≥16, ≥19, 19–64, ≥65 years	Aged ≥19 years: 62% M: 66% W: 58% (2016)^33^
Northern Ireland	Health Survey for Northern Ireland	Same as the Health Survey for England Asked of all aged ≥16 years, reported for ≥19 years	Aged ≥19 years: 55% M: 61% W: 51% (2016–2017)^66^
Scotland	Scottish Health Survey	Same as the Health Surveys for England and Northern Ireland with two exceptions: single item on occupational activity and a greater number of sport and exercise activities prompted Asked of all aged ≥16 years Included annually	Aged ≥16 years: 65% M: 71% W: 60% (2017)^34^
Wales	National Survey for Wales	7-day recall period Specific daily durations of walking, moderate, vigorous intensity activity Only asked of a subsample in some survey years Asked of all aged ≥16 years	Aged ≥16 years: 54% M: 57% W: 51% (2016–2017)^67^

M, men; W, women.

The HSNI and the HSE use identical questionnaires.^16 17^ The SHeS differs from them only on occupational activity and the number of sports prompted.^18^ In England, the ALS is structured differently in terms of how activities are reported and does not cover occupational activity.^21^ The NSW questionnaire is the only one to use a 7-day as opposed to 4-week recall period and does not ask about specific activities.^20^ All surveys distinguish between moderate and vigorous intensity activities, enabling recommendation compliance to be calculated for those who do a combination of PA intensities. In general, MVPA questions are repeated annually (see online supplementary table 1). The two exceptions are the HSE and HSNI that include their full questionnaire periodically, but include a variation of the International Physical Activity Questionnaire Short Form (IPAQ-short) in intervening years.^16 17^ All surveys ask the questions of those aged ≥16 years, although only the HSE and HSNI report separately on those over 19 years, which is the age range that the recommendation applies to.^16 17^

10.1136/bjsports-2018-100355.supp1 Supplementary data

#### Children and young people

As shown in table 3, considerable variation exists in the methods and questionnaires used to measure the percentage of children meeting the MVPA recommendation (60 min daily). The SHeS, HSE and ALS: Children and Young People survey use relatively long and detailed domain-specific questionnaires,^16 18 22^ while the YPBAS,^23^ the CHS^19^ and the HBSC surveys^24–26^ use similar variations of single-item questionnaires asking respondents to indicate on which days they achieved 60 min of MVPA. The NSW uses an extended version of this single item, asking for a summary duration of MVPA for each day of the previous week.^20^ The age ranges differ between surveys, and no surveys include young people 16–18 years old in their prevalence estimates. Parents usually proxy report for children under 10 years. The questionnaires have barely changed in the last decade, but their frequency of inclusion in the survey varies (see online supplementary table 2).

10.1136/bjsports-2018-100355.supp2 Supplementary data

**Table 3:** Overview of the UK national surveys that measure compliance with the child MVPA recommendation

Country	Survey	Current questionnaire	Latest prevalence estimate
England	Health Survey for England	7-day recall period with activities reported on specific days Domain specific, including activity at school Asked of all 2–15 years, proxy report up to age 12 Children 2–4 years old analysed separately against the under 5s recommendation	Children 2–4 years old: 9% B: 10% G: 9% Children 5–15 years old: 21% B: 24% G: 18% (2015)^27^
	Active Lives: Children and Young People Survey	Adaptive recall period 7 days/4 weeks to suit frequency Activities in past week reported on specific days Asked of years 1–11 (approximately 5–15 years) Questionnaire adapted to age of child Supplementary information from parents and teacher questionnaires	Children 5–16 years old: B: 20% G: 14% (2017/2018)^68^
Northern Ireland	Young Persons’ Behaviour and Attitudes Survey	Single-item question asking how many days in last 7 days undertaken ≥60 min MVPA Also questions to give greater detail on specific activity participation over 7-day/12-month time period Asked of children 11–16 years old	Children 11–16 years old: 13% B: 17% G: 8% (2016)^69^
	Continuous Household Survey	Single item on time spent in other sport and exercise activities per day Unanchored recall period (‘on average’) Detailed active travel questions not included in prevalence estimate Asked of school children aged 4–19 years, parental proxy report for younger children	Primary school: 39% Post-primary school: 26% (2016/2017)^70^
Scotland	Scottish Health Survey	7-day recall period, with activities reported on specific days Domain specific, including activity at school Asked of all 2–15 years, parent proxy report for younger children Headline figures reported for children 5–15 years old (and 4-year-olds at school) No reporting on the under 5s recommendation	Children 5–15 years old: 33% B: 36% G: 31% (2017)^71^
Wales	National Survey for Wales	Duration of any MVPA reported for each day in last 7 days Proxy report for all ages (only asked of those aged 3–7) from parent	Children 3–7 years old: 51% B: 55%, G: 47% (2016–2017)^67^
England, Scotland, Wales	Health Behaviour in School-Aged Children	Single-item question where respondents report the days in last seven when undertaken ≥60 min MVPA Children 11, 13, 15 years old, school-based survey	15%–19% B: 20%–22% G: 11%–15% (2014)^24–26^

B, boys; G, girls; MVPA, moderate-to-vigorous physical activity.

The only survey to report on the MVPA recommendation in the under 5s is the HSE.^27^ Parents of children in this age group are asked the same questionnaire as for children 5–15 years old. Compliance with the recommendation is monitored by setting the threshold at 180 min per day rather than 60. However, the aerobic recommendation for the under 5s age group includes ‘light’ intensity PA, whereas the HSE only asks about MVPA. Thus, information on ‘light’ intensity PA, which should be included in the prevalence estimate for under 5s, is not collected.

### Measurement of muscle strengthening activity

The HSE, SHeS and the HSNI can all estimate the percentage of adults and older adults undertaking muscle strengthening activity on at least 2 days per week.^16–18^ This is based on the reported frequencies of sport and exercise activities which are considered to be muscle strengthening such as climbing, rowing and swimming (see Strain *et al* 28). The ALS and the NSW do not measure this recommendation.^20 29^

Reporting on the percentage of the population meeting the muscle strengthening recommendation has been inconsistent. The HSE last reported relevant figures in 2012 (34% men and 24% women),^30^ the SHeS in 2015 (30% men and 25% women)^31^ and the HSNI in 2013/2014 (25% men and 14% women).^32^ The HSE and SHeS have since reported on the percentages of the population meeting both the MVPA and the muscle strengthening recommendations in 2016/2017 (HSE: 31% men, 23% women; SHeS: 30% men, 25% women).^33 34^ Given that only 1% of the population meet the muscle strengthening but not the MVPA recommendation,^33 34^ these figures are reasonable estimates of the percentage of the population meeting the recommended frequency of muscle strengthening exercise.

Children and young people are recommended to undertake vigorous intensity activities, including those that strengthen muscle and bone, at least 3 days a week. These activities are not specifically assessed in any of the UK surveys, meaning national prevalence has not been estimated.

### Measurement of balance and co-ordination

The older adult recommendations include balance and co-ordination improving activities on at least 2 days per week for those at risk of falls. The HSE and SHeS estimate the proportion of all adults ≥65 years meeting this recommendation, although the surveys are not designed to identify individuals who are ‘at risk of falls’.^16 18^ This is based on reported participation in balance and co-ordination improving activities such as dance, martial arts, tai chi and a wide range of sports. The HSNI could use the same method to derive estimates, but have not yet published these results.^17 35^ The ALS and NSW do not measure this recommendation.^20 21^

In the HSE’s and SHeS’ annual reports, balance and co-ordination improving PA is rarely reported. Strain *et al* used SHeS data from 2012 to 2014 to estimate that 19% of older men and 12% of older women in Scotland met the balance and co-ordination recommendation.^28^ In 2016, the HSE reported the prevalence by MVPA level: 27% of those ≥65 years meeting the MVPA recommendation also undertook the recommended level of balance and co-ordination activities. Only 11% of the ‘low/some activity’ group (30–149 min/week) met the balance recommendation, while 2% of the ‘inactive’ group (<30 min/week) did.^33^

### Measurement of sedentary behaviour

Table 4 and online supplementary table 3 present the questions used to report on the SB of adults in the four national surveys over the last decade. Empirical work over the past two decades has reported SB to be associated with a host of negative cardiometabolic health outcomes and premature mortality.^36 37^ In line with this evidence, advice to reduce SB has been included in the CMO PA guidelines since 2011. SB questions were included in surveillance infrequently across all age groups, but were less common in children’s questionnaires than adults’. For example, the NSW and its predecessor, the Welsh Health Survey, included SB questions only once for adults and never for children. The SHeS has the most consistent measurement of SB for both adults and children, asking the same questions annually since 2012.

10.1136/bjsports-2018-100355.supp3 Supplementary data

**Table 4:** Questions on sedentary behaviour for adults and children in national surveys since 2008

Country	Survey	Population	Year										Most recent prevalence statistics
			2017	2016	2015	2014	2013	2012	2011	2010	2009	2008	
England	Health Survey for England	Adults	?	B	E	E	E	B	X	X	X	B†	Mean: 4.7 hours/weekday^33^‡
		Children	?	X	D	X	X	D	X	X	X	D†	Mean: 3.0 hours/weekday^27^
Northern Ireland	Health Survey for Northern Ireland	Adults	B	E	X	B	?	X	X				44% adults report ≥4 hours/weekday^35^
	Young Persons’ Behaviour and Attitudes Survey	Children		S			S			S			Not reported
Scotland	Scottish Health Survey	Adults	A	A	A	A	A	A	X	G	X	G	Mean: 5.2 hours/weekday^34^‡
		Children	C	C	C	C	C	C	X	X	X	G	Mean: 3.4 hours/weekday^34^
	Welsh Health Survey*	Adults			F	X	X	X	X	X	X	X	None published
		Children			X	X	X	X	X	X	X	X	N/A
Wales	National Survey for Wales	Adults	X										N/A
		Children	S										81% report ≥2 hours screen time/weekday^67^
England, Scotland, Wales	Health Behaviour in School-Aged Children	Children				S				S			61%–68% report watching TV/DVDs for ≥2 hours/weekday^24–26^

Surveys crossing successive years (eg, 2015–2016) are reported under the second year. Blank cells indicate survey not conducted in that year or years when the National Survey for Wales did not include questions on health. Questionnaires for adults apply to those aged 16+, questionnaires for children apply to those aged 2 to 15.

Key (see online supplementary table 3 for full description of questions): A: sum of behaviours (work; TV; other (not TV not work)), long recall period; B: sum of behaviours (work (sit and stand); TV; other (not TV not work)), long recall period; C: sum of behaviours (TV; other (not TV, not school)), unanchored recall period; D: sum of behaviours (TV; other (not TV, not school)), previous week recall period; E and F: single-item direct measure, previous week recall period; G: single-item proxy measure (TV), long recall period (previous 4 weeks); S: asked questions about screen time, which cannot be directly ascribed to sitting; X: no questions asked about SB; ? indicates documentation not yet published/not clear from documentation; N/A, not applicable.

*Included in table as it was one of a number of surveys to be combined into the National Survey for Wales.

†Additionally, piloted device-based measurement, using hip-worn ActiGraph (not a postural measure of sitting).

‡Sitting time at work/school not included in these estimates.

The type of SB questionnaires has been consistent, falling into two broad groups. One group of single-item questions about total sitting time (reported in hours and minutes), with a previous week recall period of weekdays only (a version of the IPAQ-short),^38^ are only asked of adults. The other group, asked of both adults and children, includes composite measures of behaviours: work; TV viewing; other, described as any non-work (adults) or school (children) otherwise not reported. Although adults were sometimes asked about work in these composite questions, children were never asked an equivalent question about sitting time at school. For adults, the recall period of the questions was the last 4 weeks. For children, it was either unanchored (ie, a typical week) or for the previous week. Two of these SB behaviours (TV and other) were asked separately for week and weekend days, whereas questions about work SB were asked about the work day. As with the MVPA questions, parents usually proxy report for children under 10 years.

Reporting of SB results in the annual reports from the national surveys is even more sporadic than its measurement. For example, the SHeS has included SB questions every year since 2012, but only reported on them in the main survey report in 2012 and 2015. Additionally, the HSE and SHeS have only reported on leisure time SB, and did not include time spent sitting at work. As the 2011 guidelines do not provide a recommended threshold for sitting, surveys can only report population estimates of time spent sitting. Other surveys use different metrics, such as the proportion that report sitting or watching TV for more than a given number of hours per day, for example, HSNI (adults), NSW (children) and HBSC (children).

## Discussion

### Summary of current UK surveillance

Surveillance of PA and SB in the UK is complex and fragmented, undertaken separately in each of the four home nations, and across multiple surveys in each country that cover different age groups. Although most of the PA recommendations are covered by at least one of the surveys, the distribution of coverage is variable. Surveys rarely assess children under 5 for compliance to their age group–specific recommendations. Despite slight differences in the recommendations for adults and older adults, estimates for these two groups are not reported separately. Some aspects of the adult and older adult recommendations are not currently assessed in some home nations; for example, Wales does not assess SB, muscle strengthening or balance. The child muscle and bone strengthening recommendation is not measured by any nation.

As we move forward with the forthcoming 2019 recommendations, it is timely to consider whether the UK PA and SB surveillance system can be improved. It is important to consider potential risks and benefits for change, and alternative approaches to the existing surveys. The final wording of the 2019 recommendations will undoubtedly influence surveillance decisions.

### Validity of survey questions

Validation of the questions used in UK surveillance is limited. The current HSE adult MVPA questions, also used in the HSNI, performed comparably with other self-report instruments when compared with uni-axial waist-worn accelerometers (correlation coefficients 0.32–0.42).^39 40^ The questionnaire also produced lower prevalence estimates than the IPAQ-short by ~10%–20%, although the magnitude of this difference across demographic categories was similar.^41^ The mean daily minutes derived from a previous, but comparable, version of the SHeS child MVPA questionnaire were, on average, 122 min per day higher than those derived from uni-axial waist-worn accelerometers.^42^

Regarding SB, a recent large validation study of older adults (n=700) used the TASST framework to assess the effect of two dimensions (type of assessment n=6, recall period n=3) in a systematic manner (in a 6×3 grid, testing 18 combinations) against a device-derived postural measure of SB.^43^ This methodology allowed for generalisable statements to be made as to the optimal method of assessing self-reported SB. Measurement was poor for all combinations, and the authors recommended that a correction factor should be added to the self-reported SB to adjust the population mean value. In general, questions asking about SB as a sum of time spent in individual behaviours, as commonly used in national surveys, were the type of assessment that performed worst. A single question, a visual analogue scale of the proportion of the waking day spent sitting (eg, online supplementary figure 1), fared best in terms of precision and feasibility (missing data or survey non-response).^43^

10.1136/bjsports-2018-100355.supp4 Supplementary data

### Do differences in questions affect prevalence estimates?

The child MVPA questions have differed in terms of assessing whether a child has undertaken ≥60 min of MVPA on every day in the last week (HSE, HSNI, SHeS from 2017) or achieved an average of 60 min across the week (SHeS until 2016). Such inconsistent interpretations lead to an approximate difference of 30% in prevalence estimates.^44^

For adult MVPA, occupational behaviour is often assessed separately from other PA and SB, making it difficult to calculate total PA and SB volume estimates. The HSE and HSNI use multiple questions to derive total minutes of adult MVPA at work per week, whereas the SHeS uses a single question. This makes a substantial difference to the estimated total weekly minutes,^45 46^ but unpublished work suggests it may not affect estimates of the percentage meeting the recommendations.^47^ Regarding SB, the HSE and HSNI ask a single question about time spent both sitting and standing at work, meaning occupational SB cannot be assessed separately. Although occupational SB is questioned separately in the SHeS, it is not reported as part of total sitting time. Many working age adults spend a considerable part of their working day sitting.^48^ Reporting selectively on leisure time SB can may be misleading and, compared with total sitting (work and leisure), can lead to distorted estimates of SB distribution in the population.^34 49^ Comprehensive assessment of SB across the whole day/all domains should also apply to children in future surveillance.

### Harmonisation of questionnaires

The large variation in questionnaires, administration methods and sample populations in UK surveillance hinders cross-national comparisons. This point was emphasised in the recent WHO Global Action Plan for PA^50^ and is all the more important given the part-devolved, part-reserved nature of governance in the UK. The four CMOs overcame this organisational structure to jointly present the 2011 PA recommendations and will do again for the forthcoming 2019 update. We challenge professionals and academics involved in health surveillance to do the same as comparisons between nations are undermined if differences in the survey methods cause greater differences in prevalence than any likely true difference in population PA levels. An important counterpoint to harmonising measurement across countries/surveys is that trend data are also critical to inform and evaluate policies, meaning there has to be a strong rationale to depart from the methods used in previous surveys. As the 2019 recommendations could require trend-disrupting changes such as the removal of the 10 min minimum bout duration,^51^ the discussion about methods harmonisation is extremely timely.

The adult MVPA questions of the HSE and HSNI questionnaires are directly comparable, and they have only minor differences to the SHeS. These surveys are also those with the longest running trend data (see online supplementary table 1 for trends since 2008). However, they are much longer than the NSW questionnaire, and space is at a premium across all surveys. A possible solution would be to include the HSE/HSNI questionnaire once every 4 years in SHeS and HSNI, with the existing questionnaires in the intervening years. Yet, this solution does not suit the SHeS because of the need to pool consecutive years of data to generate local authority level estimates.

Another alternative is the IPAQ-short, as similar questions have been used in all countries but Scotland (see online supplementary table 1). However, this would not provide domain-specific information. The Global Physical Activity Questionnaire (GPAQ) is another alternative,^52^ but would result in the loss of the muscle strengthening and balance prevalence estimates which rely on the detailed sport and exercise questions.^53^ An advantage of these two questionnaires is that they are the most commonly used globally,^8^ enabling wider comparisons.

For child MVPA, variations of the HBSC questionnaire have been used in all countries except Scotland (see online supplementary table 2), but again this does not provide domain-specific information. The recent change in response categories to the SHeS questionnaire has meant that comparable estimates with the HSE may be derived, but the questionnaires are much longer than those currently used in other surveys, meaning that their adoption is unlikely.

Changing the muscle strength and balance questions may be something policy-makers are willing to consider, given that trend data on these recommendations have not been widely integrated into policy-making. However, the UK is leading international surveillance in this area: only two out of 114 other countries’ main national surveys specifically assess muscle strengthening, and none assess balance.^53^ It would therefore be regressive to lose the ability to monitor prevalence against these recommendations.

Moving to a single SB question assessing the whole day using a visual analogue scale is worth considering (see online supplementary figure 1), as the inconsistent nature of SB monitoring and reporting across all surveys means that policy-influencing trend data have not yet been established. A single question would reduce pressure on inclusion constraints for surveys, and other policy-directed questions (eg, context of SB) could be asked additionally in each survey as required.

In summary, there are costs and benefits to every potential solution. Survey funders, managers and researchers need to be involved in any decisions that are made to ensure that the views of all stakeholders are considered.

### Alternatives to questionnaire measurement

The recent WHO Global Action Plan on PA advocated the development and testing of new technologies for PA surveillance.^50^ In the UK, hip-worn accelerometers were trialled once for a subsample of the 2008 HSE, but were not adopted into national surveillance.^39^ Internationally, several national surveys (eg, USA^54^) incorporate device-based measurement of PA. In addition, large-scale accelerometry data have been collected in the UK among both adults (UK Biobank^55^ and 1970 British Birth Cohort^56^) and children (the Growing Up in Scotland Study^57^ and Millennium Cohort Study^58^).

Estimates for total time spent in MVPA and SB derived from accelerometers are more accurate than those from questionnaires.^43 59^ However, widespread adoption in surveillance requires consideration of practical concerns, including investment in processes, compliance and understanding how adoption of newer technology in different survey years will influence trend data.^54 56 60^ No device can measure all aspects of the PA recommendations. Preferred devices and wear locations are different for measuring MVPA and SB.^54 61^ Furthermore, no device can currently quantify muscle strengthening and balance activity.

Grip strength is an objective measure of muscle strength that has previously been used in the 2005 HSE.^62^ As a quick and straightforward measure that correlates well with mortality and cardiovascular disease risk,^63 64^ it may be suitable for re-inclusion in the future. However, it is a measure of fitness rather than engagement in the behaviours themselves.

In general, these measures do not assess context,^15^ which may be of policy interest. Conceptual differences in what device-based and self-report methods assess mean that recommendations derived from self-reported instruments (ie, 150 min MVPA) may not be appropriate for device-based measurement.^54^

The growth and popularity of wearable technologies to track activity and health may provide a feasible mechanism for assessing PA and SB, potentially through consented access to data stored in respondents’ own devices. Yet, what information such monitors actually capture and output varies and there are potential biases due to unequal distribution in ownership of such devices across the population (eg, greater ownership among younger and more active individuals).^65^

### Strengths and limitations

This is the first comprehensive review to describe PA and SB surveillance in the UK and will help initiate a meaningful discussion on future surveillance possibilities. This review will be of use to those interested in PA and SB surveillance within the UK and beyond. These issues are increasingly important to understand as the Go-PA! country cards and Active Healthy Kids Report Cards initiatives grow.

There are a number of limitations to the present work. The non-systematic nature of the literature search means that it is possible that some surveys have been overlooked. However, the approach used was the only realistic method to employ given the ‘grey’ nature of the literature. We were also limited by the availability of documentation: not all surveys had comprehensive methodological documentation, particularly for previous iterations. Also, the current questions may be subject to change in the near future and/or do not represent usual circumstances. Where this was a concern, we tried to contact the survey co-ordinators/administrators, although this was not always possible. Our framework of reviewing surveillance methods against the existing age-grouped recommendations meant that we were unable to explore whether there are specific surveillance issues relating to other population groups such as those with disabilities and/or chronic conditions. Finally, our work relates mostly to the 2011 UK PA recommendations. New recommendations will be published in 2019 that may raise new issues and resolve some of those highlighted. However, this work provides the necessary background to inform those decisions, as it is highly probable that the same surveys will be central to PA and SB surveillance across the UK. This comprehensive summary of the numerous inter-related issues should enable a UK-wide strategy to be developed.

### Recommendations

Our results have highlighted the ‘patchwork quilt’ nature of PA and SB surveillance in the UK. With the forthcoming 2019 update in mind,^10^ we recommend a calculated move towards harmonisation of questionnaires and, where possible, survey methods. We have presented a number of self-report options that should be considered:

- Harmonising to one of the existing questionnaires across all surveys that optimises the maintenance of current trend data;
- Introducing an internationally used questionnaire such as the IPAQ-short or GPAQ for adult MVPA while retaining a method of assessing muscle strengthening and balance activities; and
- Introducing a single-item question for SB using a visual analogue scale response.

We have also presented some of the advantages and disadvantages of these suggestions. It will be important that funders, survey managers and researchers take any decisions jointly. Given the competing pressures on national surveys, the eventual outcome may not be the optimal method that one would recommend for a large-scale research study.

We also recommend that the introduction of device-based measures is considered and that the practical and technical barriers specific to their introduction into UK surveillance are established. We suggest that the use of wearable trackers is considered at an early stage, putting the UK at the forefront of developments in PA and SB surveillance.

**What are the new findings?**

- Monitoring the percentages of people who meet the physical activity recommendations in the UK is complex, and is undertaken separately in each of the home nations, using multiple surveys that cover adults and children separately.
- There is no part of the recommendations for which comparable estimates can be calculated across all four home nations.
- We recommend that the costs and benefits of harmonising the existing questionnaires are considered, along with the potential introduction of device-based measures.
