# Supplementary material for: How are we measuring physical activity and sedentary behaviour in the four home nations of the UK? A narrative review of current surveillance measures and future directions
Source: Br J Sports Med. 2019 May 22;54(21):1269–76. doi: 10.1136/bjsports-2018-100355 (PMC7116237; doi:10.1136/bjsports-2018-100355)
Supplement: Supplementary data [file bjsports-2018-100355supp001.pdf]

Supplementary Table 1 Questions on compliance to the 2011 adult MVPA recommendation in national surveys since 2008.

Country	Survey	Year										Description of alternative method 'B'
		2017	2016	2015	2014	2013	2012	2011	2010	2009	2008	
England	Active Lives Survey[1]	A	A	B	B	B	B	X	X	X	X	Pre-2015, it was the Active People Survey. Telephone administered and different questionnaire. Pre-2012, the questions only asked about sport participation.
	Health Survey for England[2]	B	A	B	B	B	A	X	X	X	A	A variation on the IPAQ short included on years when main questionnaire is not. Note: Accelerometry piloted in a sub-sample in 2008.
Northern Ireland	Health Survey for Northern Ireland[3]	?	A	B	X	A	A	B	B			In 2015, IPAQ short was used. In 2010/11, a 7-day recall questionnaire was used.
Scotland	Scottish Health Survey[4]	A	A	A	A	A	A	A	A	A	A	Minor changes made in 2012 but trends can be calculated back to 2008.
Wales	Welsh Health Survey[5]			A	X	X	X	X	X	X	X	The Welsh Health Survey was merged into the National Survey for Wales in 2016. In 2015, the Welsh Health Survey asked half the sample the questions now included in the National Survey for Wales. Prior to that, the Welsh Health Survey measured MVPA but not in a way that enabled compliance to the 2011 recommendation to be estimated.
	National Survey for Wales[6]	A	A									

MVPA: moderate-to-vigorous physical activity; IPAQ: International Physical Activity Questionnaire. A: Used a comparable method (with tolerance to minor questionnaire changes) to those described in Table 2. B: Used an alternative method to measure compliance to the 2011 adult MVPA recommendation (descriptions included in table). X: No measure of meeting the 2011 adult MVPA recommendation included in the survey. Blank: Survey not run. ? indicates documentation not yet published.

Supplementary Table 1 references

1 Sport England, TNS BMRB. Active People Survey 5-7 Technical Report 2013. Available from: https://www.sportengland.org/media/3388/active-people-survey-6_technical-report_final.pdf.
2 UK Data Service. Health Survey for England 2018. Available from: beta.ukdataservice.ac.uk/datacatalogue/series/series?id=2000021 Archived at: http://www.webcitation.org/72e444sR1.
3 Department of Health. Health Survey Northern Ireland questionnaires. 2016. https://www.health-ni.gov.uk/publications/health-survey-northern-ireland-questionnaires Archived at: http://www.webcitation.org/7315vtYHR
4 Scottish Government. Scottish Health Survey: Reports and publications 2018. Available from: https://www.gov.scot/Topics/Statistics/Browse/Health/scottish-health-survey/Publications Archived at: http://www.webcitation.org/72e56q8aS.
5 Welsh Government. Welsh Health Survey: Past releases 2016. Available from: https://gov.wales/statistics-and-research/welsh-health-survey/?tab=previous&lang=en Archived at: .
6 Welsh Government. National Survey for Wales Design and Methodology 2018. Available from: http://gov.wales/statistics-and-research/national-survey/design-methodology/?lang=en Archived at: http://www.webcitation.org/72NnN7RpJ.
