# Supplementary material for: How are we measuring physical activity and sedentary behaviour in the four home nations of the UK? A narrative review of current surveillance measures and future directions
Source: Br J Sports Med. 2019 May 22;54(21):1269–76. doi: 10.1136/bjsports-2018-100355 (PMC7116237; doi:10.1136/bjsports-2018-100355)
Supplement: Supplementary data [file bjsports-2018-100355supp002.pdf]

Supplementary Table 2 Questions on compliance to the 2011 child MVPA recommendation in national surveys since 2008.

Country	Survey	Year										Description of alternative method 'B'
		**2017**	**2016**	**2015**	**2014**	**2013**	**2012**	**2011**	**2010**	**2009**	**2008**	
England	Health Survey for England[1]	?	X	A+B	X	X	A	X	X	X	A	In 2015, 13-15 year olds were asked the Health Behaviour in School-Aged Children questions as part of the self-administered questionnaire. In 2008, accelerometry was piloted on a subsample of those over 4 years of age.
	Active Lives: Children and Young People[2]	A										
Northern Ireland	Young Persons' Behaviour and Attitudes Survey[3]		A			A			A			
	Continuous Household Survey[4]	?	A	A	A	A	X	X	X	X	X	
Scotland	Scottish Health Survey[5]	A	A	A	A	A	A	A	A	A	A	In 2017, the responses to the questionnaire were amended so that the durations of activities were reported on specific days in the last week, rather than the total frequency/average session duration.
Wales	Welsh Health Survey[6]			A	A	A	A	A	A	A	A	Very similar questions used in the Welsh Health Survey for parents of 4-12 year olds but it was self-administered not interviewer-led.
	National Survey for Wales[7]	A	A									
England, Scotland,	Health Behaviour in School-Aged				A				A			

Wales	Children[8]

MVPA: moderate-to-vigorous physical activity. A: Used a comparable method (with tolerance to minor questionnaire changes) to those described in Table 3. B: Used an alternative method to measure compliance to the 2011 child MVPA recommendation (descriptions included in table). X: No measure of meeting the 2011 child MVPA recommendation included in the survey. Blank: Survey not run. ? indicates documentation not yet published.

Supplementary Table 2 references

1 UK Data Service. Health Survey for England 2018. Available from: beta.ukdataservice.ac.uk/datacatalogue/series/series?id=2000021 Archived at: http://www.webcitation.org/72e444sR1.
2 Sport England. The Active Lives: Children and Young People Survey - Questionnaire content. London, UK: Sport England; 2018. https://www.sportengland.org/media/13277/active-lives-children-and-young-people-survey-questionnaire-overview.pdf Archived at: http://www.webcitation.org/7315mwYvd
3 Department of Health. Young persons behaviour and attitudes surveys 2014. Available from: https://www.health-ni.gov.uk/publications/young-persons-behaviour-and-attitudes-surveys.
4 Northern Ireland Statistics and Research Agency. Continuous Household Survey documents. 2017. https://www.nisra.gov.uk/publications/chs-survey-documents
5 Scottish Government. Scottish Health Survey: Reports and publications 2018. Available from: https://www.gov.scot/Topics/Statistics/Browse/Health/scottish-health-survey/Publications Archived at: http://www.webcitation.org/72e56q8aS.
6 Welsh Government. Welsh Health Survey: Past releases 2016. Available from: https://gov.wales/statistics-and-research/welsh-health-survey/?tab=previous&lang=en Archived at: .
7 Welsh Government. National Survey for Wales Design and Methodology 2018. Available from: http://gov.wales/statistics-and-research/national-survey/design-methodology/?lang=en Archived at: http://www.webcitation.org/72NnN7RpJ.
8 Currie C, Van der Sluijs W, Whitehead R, et al. Health Behaviour in School-Aged Children 2014 Survey in Scotland National Report. University of St Andrews, UK: Child and Adolescent Health Research Unit (CAHRU); 2015. http://www.cahru.org/content/03-publications/04-reports/hbsc_nr14_interactive_final.pdf Archived at: http://www.webcitation.org/72NllUq7q
