# Supplementary material for: How are we measuring physical activity and sedentary behaviour in the four home nations of the UK? A narrative review of current surveillance measures and future directions
Source: Br J Sports Med. 2019 May 22;54(21):1269–76. doi: 10.1136/bjsports-2018-100355 (PMC7116237; doi:10.1136/bjsports-2018-100355)
Supplement: Supplementary data [file bjsports-2018-100355supp003.pdf]

Supplementary Table 3 Self-report questions used in U.K. national surveys to report SB in adults and children described in terms of the TASST framework[1]

The TAxonomy of Self-reported Sedentary behaviour Tools (TASST)

**Domain name**	**Type of Assessment**	**Recall Period**	**Temporal Unit**	**Assessment Period**
A[1]	1.2.2.1 Sum of Behaviours: work; TV; other (not work, not TV).	2.3 longer (previous 4 weeks)	3.1 day	4.3 both week and weekend days. *NOTE: work asked for work (not week) days*
B[2,3]	1.2.2.1 Sum of Behaviours: work; TV; other (not work, not TV). *NOTE for work, asked about sitting and standing in the same question*	2.3 longer (previous 4 weeks)	3.1 day	4.3 both week and weekend days. *NOTE: work asked for work (not week) days*
C[1]	1.2.2.1 Sum of Behaviours: TV; other (not school, not TV).	2.4 unanchored (asks about an average week)	3.1 day	4.3 both week and weekend days.
D[2]	1.2.2.1 Sum of Behaviours: TV; other (not school, not TV).	2.2 previous week	3.1 day	4.3 both week and weekend days.
E[2,3]	1.1.1 Single item direct measure (i.e. total time spent sitting)	2.2 previous week	3.1 day	4.3 both week and weekend days.
F[4]	1.1.1 Single item direct measure (i.e. total time spent sitting)	2.2 previous week	3.1 day	4.1 week days only.
G[1]	1.1.2 Single item proxy measure (TV)	2.3 longer (previous 4 weeks)	3.1 day	4.1 week day only

Question used in: [1] the Scottish Health Survey; [2] the Health Survey for England; [3] the Health Survey for Northern Ireland; [4] Welsh Health Survey.

Reference for Supplementary Table 3

1 Dall PM, Coulter EH, Fitzsimons CF, et al. TAxonomy of Self-reported Sedentary behaviour Tools (TASST) framework for development, comparison and evaluation of self-report tools: content analysis and systematic review. *BMJ Open*. 2017;7:e013844-e. doi: 10.1136/bmjopen-2016-013844
