# Supplementary material for: How are we measuring physical activity and sedentary behaviour in the four home nations of the UK? A narrative review of current surveillance measures and future directions
Source: Br J Sports Med. 2019 May 22;54(21):1269–76. doi: 10.1136/bjsports-2018-100355 (PMC7116237; doi:10.1136/bjsports-2018-100355)
Supplement: Supplementary data [file bjsports-2018-100355supp004.pdf]

BMJ Publishing Group Limited (BMJ) disclaims all liability and responsibility arising from any reliance placed on this supplemental material which has been supplied by the author(s)

Supplementary Figure 1. Example of single direct item of sitting asked as a visual analogue scale, for a previous week recall period (reproduced from [1])

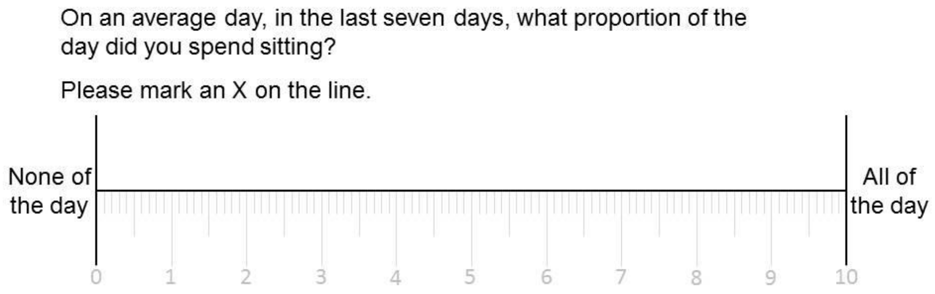

Reference for Supplementary Figure 1

1 Chastin SFM, Dontje ML, Skelton DA, et al. Systematic comparative validation of self-report measures of sedentary time against an objective measure of postural sitting (activPAL). *IJBNPA*. 2018;15:21. doi: 10.1186/s12966-018-0652-x
